# Trust as a Driver of Pro-Ecological Behaviour: The Power of Experts and Interpersonal Networks in a Low-Trust Context

**DOI:** 10.3390/bs16040511

**Published:** 2026-03-29

**Authors:** Velina Hristova, Kaloyan Haralampiev, Ivo Vlaev, Sonya Karabeliova

**Affiliations:** 1 Institute for Population and Human Studies, Bulgarian Academy of Sciences, 1113 Sofia, Bulgaria; 2 Faculty of Philosophy, Sofia University “St. Kliment Ohridski”, 1504 Sofia, Bulgaria; k_haralampiev@phls.uni-sofia.bg; 3 Center for Behavioural and Implementation Science Interventions, Yong Loo Lin School of Medicine, National University of Singapore, Singapore 117597, Singapore; vlaev@nus.edu.sg

**Keywords:** trust, pro-ecological behaviour, Bulgaria, social trust, institutional trust, expert credibility, sustainability engagement

## Abstract

Understanding the role of trust in shaping pro-ecological behaviour is essential for advancing effective environmental communication, particularly in societies characterized by low institutional credibility. This study examines how different forms of trust—scientific, institutional, mediatic, and interpersonal—predict pro-ecological behaviour in Bulgaria, a context marked by historically low levels of social and institutional trust. Drawing on a nationally representative survey of 1008 adults, participants rated their trust in six sources of environmental information (experts, public servants, politicians, media, social networks, and friends or relatives) and reported their engagement in various ecological practices. Multiple regression analysis revealed that trust in experts was the strongest positive predictor of pro-ecological behaviour, followed by trust in friends and relatives. Trust in political institutions, media, and online social networks showed no significant associations. These findings suggest that in low-trust societies, interpersonal trust and trust in experts serve as primary drivers of ecological engagement, while institutional trust alone is insufficient to mobilize collective environmental action. The study underscores the importance of fostering both scientific credibility and community-based communication to enhance public participation in sustainability initiatives.

## 1. Introduction

Understanding the factors that motivate individuals to engage in environmentally responsible behaviour is a central concern of contemporary environmental psychology and policy research. While variables such as environmental knowledge, values, and attitudes have been extensively studied, trust has emerged as a crucial yet often underestimated determinant of pro-ecological behaviour. Trust can function as both a social and psychological mechanism, linking environmental concern to concrete action by shaping how individuals interpret information, assess risks, and decide whether to participate in collective efforts toward sustainability. Empirical evidence consistently shows that higher levels of trust are associated with stronger engagement in pro-environmental behaviour (Tam & Chan, 2018; Caferra et al., 2021; Irawan et al., 2022; Cologna et al., 2022b; Hossain et al., 2022; Grünhut et al., 2023; Joffe-Nelson et al., 2024).

The present study explores the role of trust in shaping pro-ecological behaviour in Bulgaria—a national context characterized by a historically low level of social and institutional trust. In societies where distrust in institutions and public discourse is widespread, even well-designed environmental initiatives may fail to mobilize collective engagement. This study therefore investigates specifically the degree to which citizens believe various information sources—such as experts, politicians, public servants, media, online social networks, and family or friends—when they communicate about the harmfulness of products, services, or technologies to the environment.

### 1.1. The Role of Trust in Shaping Pro-Ecological Behaviour

Trust is increasingly recognized as a pivotal factor that can either strengthen or hinder the translation of environmental concern into behavioural change (Taniguchi & Marshall, 2018; Tam & Chan, 2018; Caferra et al., 2021). Two primary dimensions are typically distinguished: social trust, or generalized trust in other people and society, and institutional trust, or confidence in authorities, governments, and other formal institutions.

#### Social Trust and Pro-Ecological Behaviour

Social trust—defined as trust in others within a society, linking individuals with each other, and reflecting social bonds (Duan et al., 2022)—plays a crucial role in fostering cooperation and collective action toward environmental sustainability. Empirical evidence from large cross-national studies (Taniguchi & Marshall, 2018; Gür, 2020; Caferra et al., 2021) consistently shows that higher levels of social trust are associated with greater engagement in pro-environmental and energy-saving behaviours. Caferra et al. (2021) found that both political and social trust positively predict individuals’ tendency to reduce domestic energy consumption across Europe, with social trust exerting a robust and significant effect across diverse contexts. This relationship reflects the idea that people who feel integrated within their communities and perceive others as trustworthy are more likely to adopt cooperative norms and act in ways that produce collective benefits. Similarly, Duan et al. (2022) demonstrated that in rural China, social trust—especially interpersonal trust among neighbours and local leaders—significantly improves conservation attitudes, reinforcing the importance of community-level relationships for ecological protection. Complementary findings by Irwin and Berigan (2013) and Gür (2020) indicate that social trust reduces free-rider tendencies and promotes voluntary environmental action by enhancing empathy, social responsibility, and belief in collective efficacy. Overall, these studies underscore that pro-ecological behaviour thrives in social environments where mutual trust and shared norms of cooperation prevail. Strengthening horizontal trust within communities can therefore amplify the social foundations of sustainability, encouraging individuals to align personal behaviour with collective environmental goals.

### 1.2. Trust in Political Institutions and Pro-Environmental Behaviour

Trust in political institutions plays a complex and sometimes contradictory role in shaping pro-environmental behaviour. While several studies have found that trust in governments and regulatory agencies generally promotes engagement in both public and private ecological actions (Cologna & Siegrist, 2020; Caferra et al., 2021, others highlight that this relationship can reverse under certain conditions. Cologna et al. (2022a) reported that trust in the Swiss government’s capacity to act on climate change was negatively associated with low-impact pro-environmental behaviours, suggesting that highly trusting individuals may feel their personal efforts are redundant when they perceive governmental action as sufficient. This finding aligns with Schwartz’s norm deactivation theory, which posits that individuals may relinquish personal responsibility when they believe capable authorities will handle environmental problems (Schwartz & Clausen, 1970; Joffe-Nelson et al., 2024). Similarly, research in outdoor and recreational contexts shows that high trust in management and regulatory agencies can sometimes reduce individual engagement, as people defer responsibility to institutional actors (Joffe-Nelson et al., 2024; Taniguchi & Marshall, 2018). Nevertheless, across broader comparative analyses, political trust tends to enhance cooperative behaviours, particularly in social-democratic contexts where governments are perceived as competent, transparent, and fair (Caferra et al., 2021). These findings collectively underscore that while political trust can strengthen policy support and compliance, its impact on individual ecological behaviour depends on whether trust reinforces shared responsibility or instead leads to its delegation.

### 1.3. Trust in Experts and Pro-Ecological Behaviour

Trust in experts—particularly in climate scientists—has consistently emerged as a central factor shaping individuals’ engagement in pro-environmental behaviour (Cologna & Siegrist, 2020). Low trust in experts, by contrast, can foster reliance on non-scientific or conspiratorial sources, thereby diminishing public engagement in collective environmental efforts. Both Cologna et al. (2022a) and Cologna et al. (2025) demonstrate that trust in climate scientists functions as a cognitive shortcut that helps individuals navigate the complexity of environmental information and make decisions under uncertainty. People who trust scientific expertise are more likely to adopt both low- and high-impact pro-environmental behaviours, including those whose mitigation potential they might otherwise underestimate, such as adopting sustainable diets. This relationship reflects broader patterns observed across studies of science communication, where trust in scientists facilitates receptivity to expert messages and enhances confidence in evidence-based solutions.

However, trust in science is not uniform across societies. Research by Kahan et al. (2012) reveals that greater scientific literacy does not necessarily translate into greater concern about climate change; rather, individuals with a higher degree of science literacy and stronger technical reasoning skills often display more polarized views, aligning their beliefs with the cultural or ideological values of their social groups. This finding underscores that trust in scientists operates within broader cultural and political frameworks that shape how evidence is interpreted. Similarly, Cologna et al. (2022b, 2025) emphasize that while most publics perceive climate scientists as trustworthy, political polarization—particularly in contexts such as the United States—can erode this confidence. A recent meta-narrative review by Fage-Butler et al. (2022) further highlights the multidimensional nature of trust in climate science, encompassing attitudinal, cognitive, affective, contextual, and communicated dimensions that jointly influence public acceptance of scientific information.

Empirical work by Anderegg et al. (2010) reinforces the legitimacy of expert authority, showing that an overwhelming majority of active climate researchers endorse the scientific consensus on anthropogenic climate change. Yet, as Joffe-Nelson et al. (2024) caution, excessively high trust can sometimes reduce individual engagement if people believe that experts and institutions are already managing the problem—a dynamic consistent with norm deactivation theory (Schwartz & Clausen, 1970). Overall, trust in experts plays a dual role: it motivates climate action by reducing uncertainty and guiding informed decisions, but when mediated by cultural identity or excessive deference to authority, it can also weaken individuals’ perceived responsibility to act.

### 1.4. Trust in Media and Pro-Ecological Behaviour

Trust in media plays a complex yet significant role in shaping pro-environmental behaviour. Arlt et al. (2011) found that in Germany, media use influenced behavioural intentions toward climate-friendly actions, but the effects varied across media types: while political television programs and combined media exposure could foster engagement in climate-related activities, print media sometimes had negative effects on willingness to change lifestyles. Similarly, Huang (2016) demonstrated that in Taiwan, attention to global warming media coverage—across television, newspapers, and online sources—positively predicted accommodating, promotional, and proactive pro-environmental behaviours, partly through enhanced environmental beliefs and self-efficacy. Han and Cheng (2020) added that media not only inform but also activate social norms: social media, more than traditional media, effectively strengthened subjective norm perceptions that encouraged pro-environmental actions. However, media effects depend strongly on trust and perception. Studies by Olausson (2018) and Schäfer and Painter (2021) highlighted that while climate journalism remains a key source of information, audiences interpret messages through diverse cognitive and cultural filters, and trust in media cannot be assumed. Moreover, Hart et al. (2015) showed that ideological orientation shapes how people process environmental news, sometimes reinforcing skepticism rather than engagement. Taken together, these findings suggest that trust in and engagement with diverse, credible media sources can foster awareness and collective efficacy necessary for climate action, yet media effects remain mediated by cognitive, ideological, and social factors that determine how messages translate into actual pro-environmental behaviour.

### 1.5. Trust in Online Social Networks and Pro-Ecological Behaviour

Ballew et al. (2015) emphasize that the relational and experiential functions of Web 2.0 technologies and social media platforms can foster environmentally responsible behaviours by enabling social learning, community building, and norm reinforcement. When users trust these online environments, they are more likely to engage in discussions, share pro-environmental messages, and adopt sustainable practices modeled by peers. Similarly, Bojanowska and Kulisz (2020) demonstrate that social media eco-marketing can effectively raise environmental awareness, particularly among women, when users perceive such content as authentic and aligned with their values. Trust also enhances participation in value co-creation: Koswara et al. (2024) found that credible corporate communication about eco-friendly products on social media promotes individuals’ intent to contribute to environmental goals. In the energy sector, Czarnecka et al. (2022) show that social media engagement strengthens perceptions of companies’ environmental responsibility and motivates green consumption choices. Broader analyses of climate discourse on Twitter (Cody et al., 2015; Dellmuth & Shyrokykh, 2023) reveal that social media not only spread awareness but also shape public opinion and non-state climate action, suggesting that trust in these platforms underpins effective climate communication and governance. Yet, as Vraga and Bode (2021) warn, misinformation and declining media literacy threaten to erode this trust, underscoring the need for credible, transparent, and evidence-based environmental communication online.

### 1.6. The Problem of Trust in the Bulgarian Context

Trust has long been identified as a structural weakness in Bulgarian society, shaped by historical legacies and persistent institutional instability. Anthropological and sociological analyses emphasize that Bulgaria maintains a continuity of distrust stretching from the socialist period into the post-socialist era (Luleva, 2021). The authoritarian past, marked by surveillance and fear of political persecution, left enduring patterns of defensive social behaviour in which distrust operates as a protective strategy. Although political repression has ended, citizens continue to experience “permanent social insecurity,” feelings of “lack of rules,” and perceptions of institutionalized injustice, which hinder the development of generalized and institutional trust (Luleva, 2021). Empirical data confirm these tendencies: levels of both social and institutional trust in Bulgaria are among the lowest in Europe (Open Society Institute—Sofia, 2018). Bulgarians exhibit relatively higher trust in immediate social circles—family and neighbours—but very low trust in political institutions, the judiciary, and NGOs. This pattern of particularized trust coexists with deep skepticism toward collective institutions, which undermines civic engagement and cooperative behaviour (Kuburic & Kuburic, 2010). Comparative studies of elites reveal a similar duality: while Bulgarian elites demonstrate relatively high trust in European institutions, their confidence in national institutions remains weak, reflecting a broader sense of disconnection between citizens and the state (Kostova, 2010). A recent illustration of this fragile trust climate emerged during the COVID-19 pandemic, when widespread uncertainty and fear revealed persistent mistrust in government, media, and even science. Despite an initial surge of confidence in authorities in the early weeks of the crisis, distrust quickly resurfaced, becoming a major obstacle to the implementation of public health measures and effective risk communication (Luleva, 2021). In this context, trust deficits present a major obstacle for pro-ecological mobilisation. Low institutional credibility and a historically grounded culture of distrust make it difficult for citizens to believe that environmental policies are fair, effective, or worth supporting, thereby limiting the translation of environmental concern into collective action.

The goal of this article is to empirically examine how different forms of trust influence pro-ecological behaviour among Bulgarian citizens. Building on the theoretical understanding that trust functions as a key social mechanism enabling cooperation and collective action, the study aims to identify which sources of trust—scientific, institutional, mediatic, or interpersonal—most effectively predict willingness to engage in environmentally responsible practices. By analysing trust in various communicators of environmental information, the research seeks to clarify how credibility perceptions shape both individual and collective pro-environmental engagement within a context of chronic institutional distrust.

## 2. Methods

### 2.1. Study Design and Sample

The study is based on a nationally representative survey of the adult population of Bulgaria (N = 1008), conducted by a professional research agency using a quantitative face-to-face interview method (CAPI) in March 2024. Data were collected through semi-structured interviews administered by trained interviewers in respondents’ homes using tablets.

The sampling design followed a stratified multistage probability approach. The sampling frame was based on the latest official statistics from the National Statistical Institute. The population was first stratified by administrative region (the 28 administrative districts) and type of settlement (capital city, regional centers, small towns, and villages). Within these strata, clusters of settlements or urban districts were selected, after which interviewers followed a random route procedure starting from predefined addresses. Within each contacted household, only one adult respondent was selected, with quotas applied for gender and age to ensure demographic balance. This procedure ensured that the final sample reflects the structure of the adult population of the country.

The questionnaire consisted of several thematic modules, including trust, pro-environmental behaviour, institutional attitudes, and socio-demographic characteristics. As a result, the average interview duration was approximately 35–40 min. The survey collected structured quantitative responses only, and no qualitative narratives or transcripts were recorded.

Quality control procedures included supervision by regional coordinators, telephone verification of 10–15% of completed interviews, and software-based validation (including GPS location checks and detection of irregular response patterns). The maximum stochastic sampling error is ±3.1% at a 50% proportion, which is standard for national surveys of this size.

The final sample consisted of 1008 adults. The sample characteristics are described in Table 1. The sample distribution closely corresponds to the population structure for gender and most age groups. A moderate underrepresentation is observed for respondents aged 70+, which is a common limitation in face-to-face surveys due to accessibility and response constraints among the oldest population groups. Of all respondents, 48.2% were men and 51.8% women. The age distribution was relatively balanced, with 14.3% aged 18–29, 16.8% aged 30–39, 19.7% aged 40–49, 18.1% aged 50–59, 17.6% aged 60–69, and 13.5% aged 70 or older. Most participants reported secondary education (59.5%), followed by higher or semi-higher (29.3%) and primary or lower (11.2%). In terms of occupation, the largest groups were workers (35.1%), employed specialists (25.3%), and retirees (22.1%). Among those reporting income, the majority (60.9%) earned between 501 and 1500 BGN per month. The ethnic composition reflected the national demographic structure, comprising 83.3% Bulgarians, 8.5% Turks, and 6.3% Roma. Settlement type was evenly distributed, with 21.5% residing in the capital, 33.4% in regional centers, 21.4% in small towns, and 23.7% in villages.

### 2.2. Measures

#### 2.2.1. Trust in Information Sources

Participants were asked to indicate the degree of trust they have in various sources when informed that a given product, service, or technology is harmful to the environment. Respondents were asked: “To what extent do you personally trust each of the following sources when they say that a certain product, service, or technology is harmful to the environment?” The question included six sources: experts, public servants, politicians, media, social networks, and friends or relatives. Responses were recorded on a four-point Likert scale ranging from 1 = not at all trust to 4 = completely trust. The scale demonstrated good internal consistency (Cronbach’s α = 0.828) (Hair et al., 2019).

#### 2.2.2. Pro-Ecological Behaviour

Pro-environmental behaviour was assessed using a set of statements covering everyday ecological practices, consumption habits, waste management, energy and water conservation, transportation, and participation in environmental initiatives. Participants rated the extent to which each statement applied to them on a four-point scale (1 = does not apply at all to 4 = fully applies).

A composite index of pro-environmental behaviour was calculated as the mean score across all items (sum of item responses divided by the number of items). One item (item 14) was reverse-coded prior to index construction. The scale demonstrated good internal consistency (Cronbach’s α = 0.833) (Hair et al., 2019).

The scale was constructed by adapting and combining items from established classifications of pro-environmental behaviour. In particular, the selection of behavioural domains was informed by the typology proposed by Kaiser et al. (2003), who identify several categories of ecological behaviour, including energy conservation, mobility and transportation, waste reduction, consumer behaviour, recycling, and social environmental actions. The conceptual structure of the scale was also guided by the synthesis of pro-environmental behavioural domains presented by Kurisu (2015), which highlights key behavioural areas such as household energy efficiency, waste and recycling, water use, sustainable consumption, transportation, and environmental activism.

Based on these frameworks, items were selected, adapted, and contextualized to reflect common everyday behaviours relevant to the Bulgarian context. The final instrument included behaviours of varying environmental impact, ranging from routine household practices (e.g., saving energy, reducing waste) to more involved civic actions (e.g., participating in environmental campaigns or initiatives). All 23 statements used in the analysis are presented in Table A1 (Appendix A).

### 2.3. Procedure and Analysis

The survey was administered by trained interviewers using a structured questionnaire. Descriptive statistics were first computed to summarize patterns of trust and behaviour. A multiple regression analysis was then conducted to examine the extent to which trust in different information sources predicts overall pro-ecological behaviour. All statistical analyses were performed using standard significance levels (*p* < 0.05).

## 3. Results

### 3.1. Descriptive Statistics

The descriptive statistics indicate clear differences in levels of trust across various information sources in Bulgaria. Respondents expressed the highest trust in friends and relatives (M = 3.83, SD = 0.90), followed by experts (M = 3.35, SD = 1.22). Trust in media (M = 2.88, SD = 1.15) and social networks (M = 2.98, SD = 1.12) was moderate, while trust in public servants (M = 2.58, SD = 1.14) and especially politicians (M = 1.94, SD = 1.07) was substantially lower. The overall mean trust score across all sources (M = 2.94, SD = 0.80) suggests generally low to moderate trust levels, consistent with Bulgaria’s well-documented trust deficit. The average pro-ecological behaviour score (M = 2.89, SD = 0.62) indicates a moderate level of engagement in environmentally responsible actions.

### 3.2. Regression Analysis: Trust and Pro-Ecological Behaviour

A multiple regression analysis was conducted to examine how trust in different information sources regarding ecological issues predicts pro-ecological behaviour (Table 2). The independent variables included trust in experts, public servants, politicians, media, social media, and friends and relatives, while the dependent variable was the pro-ecological behaviour index—calculated as the sum of respondents’ scores across the pro-environmental behaviour statements.

The regression model was significant, F(6, 738) = 16.57, *p* < 0.001, explaining 11.9% of the variance in pro-ecological behaviour (R^2^ = 0.119, Adjusted R^2^ = 0.112), indicating a modest but meaningful effect size for social perception variables.

Multicollinearity diagnostics confirmed no statistical concerns (Tolerance > 0.10; VIF < 10).

The results show that trust in experts is the strongest positive predictor of pro-ecological behaviour (β = 0.194, *p* < 0.001), followed by trust in friends and relatives (β = 0.101, *p* = 0.014). Trust in politicians, public servants, media, and social media does not significantly predict ecological behaviour.

## 4. Discussion

The findings of this study confirm that trust is a decisive factor shaping pro-ecological behaviour, even within a societal context characterized by chronic institutional distrust, such as Bulgaria. Consistent with previous research (Cologna et al., 2022a, 2025), the results demonstrate that trust in experts is the strongest predictor of environmentally responsible behaviour among Bulgarian citizens. Trust in scientific expertise appears to function as a cognitive shortcut that enables individuals to navigate the complexity of environmental information and to make decisions under uncertainty. When citizens lack detailed environmental knowledge or are exposed to conflicting messages, confidence in experts provides a reliable basis for interpreting risks and assessing the credibility of ecological claims. In this way, expert trust not only facilitates behavioural engagement but also strengthens public receptivity to scientific communication.

Equally important, trust in friends and relatives significantly predicted pro-ecological engagement, suggesting that interpersonal trust and peer influence are powerful drivers of everyday ecological practices. This finding resonates with previous research on social trust and pro-ecological behaviour (Caferra et al., 2021; Taniguchi & Marshall, 2018; Gür, 2020; Duan et al., 2022) and aligns with Irwin and Berigan’s (2013) social dilemma analysis, which demonstrated that cooperation in environmental protection depends not only on generalized trust but also on the strength of social ties and cultural context. The results for Bulgaria suggest that social trust plays a nuanced role in shaping pro-ecological behaviour, reflecting the country’s broader cultural profile of strong uncertainty avoidance, high power distance, and moderate individualism (Karabelova, 2011). In such a context, interpersonal trust tends to be concentrated within close social circles—family and friends—rather than extended to broader society or institutions. This pattern helps further explain why Bulgarians’ pro-environmental engagement is more strongly predicted by trust in relatives and peers than by institutional or generalized trust. As Irwin and Berigan (2013) noted, cooperation in social dilemmas often depends on institutional trust in collectivist cultures, whereas in more individualistic or fragmented societies, trust operates at the interpersonal level. In Bulgaria’s low-trust environment, where confidence in government, media, and other collective actors remains weak (Luleva, 2021; Open Society Institute—Sofia, 2018), informal networks may serve as alternative channels of credibility and motivation for ecological action. Strengthening such interpersonal trust and community-based initiatives could therefore be a viable strategy for fostering local-level environmental engagement.

In contrast to the significant influence of interpersonal trust, trust in political institutions, media, and online social networks did not significantly predict pro-ecological behaviour. This finding aligns with Bodor et al. (2020), who identified marked regional differences in levels of institutional trust and climate engagement across Europe. Their analysis revealed that while most Europeans acknowledge the reality of climate change and express concern about its consequences, citizens in Central and Eastern Europe—where trust in institutions is generally lower—tend to feel less personal responsibility for mitigating climate change and show weaker support for related policy measures. These patterns suggest that institutional trust alone may be insufficient to drive pro-environmental action, particularly in contexts where public confidence in authorities and media is fragile. As Schäfer and Painter (2021) note, climate journalism—the primary channel through which many citizens receive information about climate issues—is itself undergoing major transformations, with shrinking specialist reporting, the growing influence of stakeholder public relations, and the rise of digital and social media altering the credibility and perceived independence of environmental information. In such low-trust societies, environmental concern does not necessarily translate into behavioural or policy support, highlighting the importance of individual and community-level trust networks as more effective drivers of ecological engagement than formal institutional credibility.

Several additional mechanisms may help explain the observed patterns. One potential explanation is the diffusion of responsibility, a well-established phenomenon in social psychology whereby individuals are less likely to take action when responsibility is perceived to be shared among many actors (Darley & Latane, 1968). Environmental problems such as climate change are frequently perceived as large-scale collective challenges, which may encourage individuals to assume that governments, institutions, or other societal actors should take primary responsibility for addressing them. In low-trust institutional environments, such diffusion of responsibility may further weaken individual motivation to engage in pro-ecological behaviour if citizens perceive institutional actors as ineffective or unreliable.

Another possible explanatory mechanism relates to framing effects in environmental communication. Research in behavioural science and climate communication has shown that the way environmental problems are framed can significantly influence public engagement and behavioural responses (Nisbet, 2009; Spence & Pidgeon, 2010; van der Linden et al., 2015). However, framing effects may interact with trust and perceived efficacy. When individuals perceive environmental risks as severe but have limited trust in the institutions responsible for addressing them, concern may not translate into behavioural engagement. Although the present study did not directly measure framing effects or perceived efficacy, these factors may shape how environmental information is interpreted and acted upon.

The results also highlight the importance of distinguishing between national identity and community identity when considering the role of social norms in environmental behaviour. While national identity may sometimes be associated with politicized narratives or institutional distrust in post-socialist contexts, community identity is grounded in more immediate social relationships and localized networks. Research on social norms and pro-environmental behaviour suggests that individuals are particularly responsive to behavioural expectations within their immediate social environment, where comparison with similar others is most salient (Goldstein et al., 2008). Such localized normative influence can facilitate the emergence of collective behavioural standards that reinforce environmentally responsible practices.

In this respect, the findings are consistent with evidence from other low-trust contexts where community-based initiatives have successfully mobilized environmental action. For example, grassroots community groups in Bangladesh have organized collective efforts to clean and restore local waterways despite limited institutional support (BD Clean, n.d.). Similar community-driven environmental initiatives have been documented in other parts of the world (Global Citizen, n.d.), where strong local networks and shared community identities can compensate for weak institutional capacity and enable collective environmental stewardship (Pretty & Ward, 2001; Ostrom, 2000). These examples further support the argument that interpersonal trust and localized social norms can function as key mechanisms for promoting pro-ecological behaviour in societies characterized by low institutional trust.

### Practical Implications

The findings of this study highlight the crucial role of different forms of trust—particularly in experts and interpersonal networks—in motivating pro-ecological behaviour within Bulgaria’s low-trust environment. These results suggest several practical directions for improving environmental communication and public engagement.

First, scientific credibility and expert communication could be prioritized to enhance public trust in environmental messages. Environmental campaigns might benefit from featuring scientists, researchers, and other credible experts as the primary voices in communication efforts. Emphasizing independent, evidence-based perspectives could strengthen message reliability, counter misinformation, and foster greater confidence in environmental initiatives. Consistent with the psychological insights outlined by van der Linden et al. (2015), framing environmental risks as immediate, local, and personally relevant could further increase public engagement and policy support.

Second, stronger collaboration between scientific experts, journalists, and environmental communicators could improve both the accessibility and credibility of environmental information. Partnerships between media professionals and scientists can facilitate the translation of complex scientific knowledge into clear, relatable narratives that resonate with broader audiences. Such collaborative approaches may help address the growing challenges associated with fragmented media environments, misinformation, and declining public trust in traditional information sources.

Third, the results suggest that community-based and peer-driven interventions may be particularly effective in encouraging ecological engagement. Since trust in friends and relatives significantly predicts pro-environmental behaviour, initiatives that leverage interpersonal networks and social norms could help translate environmental concern into everyday behavioural practices. Examples include neighbourhood sustainability projects, local recycling initiatives, citizen-led environmental monitoring, and community clean-up campaigns. These initiatives can strengthen local social norms around environmental responsibility and make sustainable behaviours more visible, socially reinforced, and attainable. Activating descriptive and injunctive social norms—where individuals observe environmentally responsible behaviour among peers—has been shown to be an effective mechanism for promoting sustainable action (Goldstein et al., 2008).

Additionally, policymakers and environmental organizations may benefit from supporting grassroots and community-level environmental initiatives that foster cooperation and collective action. Local projects—such as community recycling programs, river and park restoration initiatives, or citizen-led environmental campaigns—can build social capital and reinforce trust within communities. In low-trust institutional environments, such grassroots efforts can serve as important catalysts for environmental engagement by demonstrating tangible examples of collective ecological action and reinforcing shared social norms around sustainability.

Finally, communication strategies could be adapted to cultural and demographic contexts to improve their resonance and impact. In a society characterized by moderate individualism and strong interpersonal bonds, messages that combine scientific accuracy with relatable narratives—emphasizing shared responsibility, community identity, and tangible benefits—could more effectively motivate behavioural change.

The study has several limitations. A primary limitation concerns its reliance on self-reported data, which may be affected by social desirability bias and potential inaccuracies in how participants perceive or report their own pro-ecological behaviours. Moreover, the cross-sectional design constrains the ability to infer causality between trust dimensions and environmental actions, as it captures associations only at a single point in time. While the sample provides valuable insights into the Bulgarian context, its specificity also limits the generalizability of the findings to other cultural or socio-political settings, particularly those characterized by different levels of institutional trust, civic engagement, or environmental awareness.

## 5. Conclusions

In summary, trust in experts and close social circles on ecological topics significantly predicts pro-ecological behaviour among Bulgarian citizens. Effective environmental interventions should therefore combine scientifically credible communication with socially resonant, peer-driven outreach, building a culture of trust that supports sustainable behaviour and environmental policy acceptance.

## Figures and Tables

**Table 1:** Sample Characteristics (N = 1008).

Variable	Category	n	% Sample	% Population	Difference	t	*p*
Gender	Male	486	48.2	48.1	0.1	0.03	0.976
	Female	522	51.8	51.9	−0.1	−0.03	0.976
Age Group	18–29 years	144	14.3	12.8	1.5	0.64	0.523
	30–39 years	169	16.8	14.4	2.4	0.96	0.339
	40–49 years	199	19.7	17.7	2.0	0.75	0.453
	50–59 years	182	18.1	17.7	0.4	0.16	0.877
	60–69 years	177	17.6	16.8	0.8	0.31	0.754
	70+ years	136	13.5	20.6	−7.1	−3.10	0.002
Education	Primary or lower	113	11.2	13.1	−1.9	−0.90	0.369
	Secondary	600	59.5	53.1	6.4	1.95	0.053
	Higher (university or postgraduate)	295	29.3	33.8	−4.5	−1.48	0.141
Place of Residence	Sofia (capital)	217	21.5	19.2	2.3	0.84	0.404
	Other urban areas	552	54.8	54.5	0.3	0.09	0.928
	Rural areas	239	23.7	26.3	−2.6	−0.91	0.362
Employment Status	Employed	608	60.3	53.2	7.1	2.17	0.031
	Unemployed	71	7.1	2.3	4.8	2.79	0.006
	Student	27	2.7	44.5	−11.9	−10.96	0.000
	Retired	223	22.1				
	Other/not specified	79	7.8				

**Table 2:** Predictors of Pro-Ecological Behaviour.

Predictor	B	SE	β	t	*p*	Partial R
(Constant)	2.098	0.101	—	20.743	<0.001	
Trust in experts	0.099	0.025	0.194	4.002	<0.001	0.146
Trust in public servants	0.018	0.031	0.033	0.572	0.568	0.021
Trust in politicians	0.051	0.030	0.089	1.736	0.083	0.064
Trust in media	−0.001	0.029	−0.001	−0.024	0.981	−0.001
Trust in social media	0.025	0.027	0.045	0.942	0.347	0.035
Trust in friends and relatives	0.070	0.029	0.101	2.457	0.014	0.090

## Data Availability

The data is available from the corresponding author upon reasonable request.

## Appendix A

**Table A1:** Pro-environmental behaviour: English translation of the items.

Items:
I prefer seasonal, locally grown food.
I buy organic food (ecological and bio products).
I read product labels with regard to their potential harm to health.
I read product labels with regard to the environmental impact of their production.
I am a vegetarian or vegan.
At home, we have separate bins for paper, glass, and household waste.
Containers for separate waste collection are located close to my home.
I try to reduce the amount of waste I generate.
I take part in campaigns for collecting bottle caps, paper, clothes, or plastic.
I participate in initiatives for cleaning and greening.
I hand over my old electrical appliances for recycling.
I turn off the lights when I am not in the room.
I turn off the water while brushing my teeth.
I throw used batteries in the general waste bin.
I have renovated my home to make it more energy efficient.
I intentionally buy products made from recycled materials.
I change my phone when a newer model appears on the market.
I use reusable shopping bags when shopping.
My household owns more than one car.
I use public transport to get to work and for everyday tasks.
I spend most of my free time in nature.
I think that it is possible to get around my town or city by bicycle.
I go to the park or mountains by car.
